# Language support for linguistic minority chronic hepatitis B/C patients: an exploratory study of availability and clinicians’ perceptions of language barriers in six European countries

**DOI:** 10.1186/s12913-017-2095-5

**Published:** 2017-02-20

**Authors:** Abby M. Falla, Irene K. Veldhuijzen, Amena A. Ahmad, Miriam Levi, Jan Hendrik Richardus

**Affiliations:** 1 Department of Public Health, Erasmus MC, University Medical Center Rotterdam, Rotterdam, The Netherlands; 2 Division of Infectious Disease Control, Municipal Public Health Service Rotterdam-Rijnmond, Rotterdam, The Netherlands; 3 Department of Health Sciences, Faculty Life Sciences / Public Health Research, Hamburg University of Applied Sciences, Hamburg, Germany; 4 Department of Health Sciences, Division of Hygiene, Preventive Medicine and Public Health, University of Florence, Florence, Italy

**Keywords:** Chronic viral hepatitis, Hepatitis B, Hepatitis C, Translating, Migrants, Linguistic minorities, Language barriers

## Abstract

**Background:**

Language support for linguistic minorities can improve patient safety, clinical outcomes and the quality of health care. Most chronic hepatitis B/C infections in Europe are detected among people born in endemic countries mostly in Africa, Asia and Central/Eastern Europe, groups that may experience language barriers when accessing health care services in their host countries. We investigated availability of interpreters and translated materials for linguistic minority hepatitis B/C patients. We also investigated clinicians’ agreement that language barriers are explanations of three scenarios: the low screening uptake of hepatitis B/C screening, the lack of screening in primary care, and why cases do not reach specialist care.

**Methods:**

An online survey was developed, translated and sent to experts in five health care services involved in screening or treating viral hepatitis in six European countries: Germany, Hungary, Italy, the Netherlands, Spain and the United Kingdom (UK). The five areas of health care were: general practice/family medicine, antenatal care, health care for asylum seekers, sexual health and specialist secondary care. We measured availability using a three-point ordinal scale (‘very common’, ‘variable or not routine’ and ‘rarely or never’). We measured agreement using a five-point Likert scale.

**Results:**

We received 238 responses (23% response rate, *N* = 1026) from representatives in each health care field in each country. Interpreters are common in the UK, the Netherlands and Spain but variable or rare in Germany, Hungary and Italy. Translated materials are rarely/never available in Hungary, Italy and Spain but commonly or variably available in the Netherlands, Germany and the UK. Differing levels of agreement that language barriers explain the three scenarios are seen across the countries. Professionals in countries with most infrequent availability (Hungary and Italy) disagree strongest that language barriers are explanations.

**Conclusions:**

Our findings show pronounced differences between countries in availability of interpreters, differences that mirror socio-cultural value systems of ‘difference-sensitive’ and ‘difference-blindness’. Improved language support is needed given the complex natural history of hepatitis B/C, the recognised barriers to screening and care, and the large undiagnosed burden among (potentially) linguistic minority migrant groups.

**Electronic supplementary material:**

The online version of this article (doi:10.1186/s12913-017-2095-5) contains supplementary material, which is available to authorized users.

## Background

Most chronic viral hepatitis infections in Europe are detected among migrants born in countries with a medium to high prevalence of hepatitis B and/or C [1]. This includes most of Africa and Asia, Central/Eastern Europe and the countries of the former Soviet Union [2, 3]. Chronic viral hepatitis B (CHB) and C (CHC) infections have a complex natural history and could require lifelong clinical monitoring and antiviral treatment [4]. People chronically infected with hepatitis B or C can also remain infectious to others and should modify or avoid certain behaviours that have a high risk of transmission [5]. These features underline the need to provide patients with information and advice about the implications of a diagnosis such as referral to specialist secondary care, diagnostic tests required, the availability of antiviral treatment, how to prevent onward transmission, contact tracing and HBV vaccination. However, research suggests that many diagnosed patients do not reach secondary care for clinical monitoring and antiviral treatment [6, 7], that language barriers are perceived to be the primary barrier to health care for viral hepatitis [8], and that immigration status is associated with not receiving treatment [9]. The asymptomatic nature as well as a lack of screening and suboptimal referral strategies means that more than 60% of people infected are unaware of their infection, undiagnosed and not in treatment [1, 10, 11]. Effective antiviral treatment for both chronic hepatitis B/C that can prevent the development of cirrhosis and hepatocellular carcinoma, and with newer direct acting anti-virals (DAAs) reporting cure rates in up to 90% of cases of chronic hepatitis C, [12] the elimination of chronic viral hepatitis a possibility in Europe [13]. This will require the continued primary prevention of new infections alongside the expansion of secondary prevention through screening and treatment.

Language barriers between linguistic minority migrants (migrants who face language barriers because they do not speak the local language) and health care professionals are reported to increase inequalities in health care via adverse effects on accessibility, quality of care, patient satisfaction, patient safety and patient health outcomes [14]. A systematic review of medical interpreter services in the United States (US) showed negative health outcomes as well as poor knowledge and understanding of diagnoses, treatment and implications of the disease, among patients who needed but did not have access to interpreter services, which resulted in inaccurate medical history-taking and missed/incorrect diagnoses [15]. Conversely, interpreters have been demonstrated to have a positive impact both on clinical outcomes and in reducing inequalities [16]. Thus, interpreters are deemed to be of benefit from both the perspectives of social justice and of evidence-based clinical medicine. Studies that examine good practice in health care for migrants recommend the provision of interpreters and/or translated materials to overcome language barriers as a means to improve patients safety, the quality of health care, medical ethical practice and patient outcomes [16, 17]. Good practice studies of viral hepatitis screening programmes among at-risk migrant populations also provide translated materials and/or interpreters to improve screening uptake, to reach more vulnerable sub-populations (those with very limited local language skills) and to raise awareness in communities at risk [18–21].

There is a distinct lack of disease-specific research however, and much of the literature about language barriers is focused on the countries of the English speaking world (the United States (US), Canada, the UK and Australia) where language proficiency is defined as limited English proficiency. We use ‘linguistic minority’ as a more appropriate term for our research in the European Union (EU), specifically in Germany, Hungary, Italy, the Netherlands, Spain and the United Kingdom (UK) These six European countries are the locations of the academic and clinical teams participating in the HEPscreen project from which this study arose ([http://www.hepscreen.eu]). They also differ considerably in their history and experience of migration as well as the health system response to diversity. The proportion of the adult population defined as foreign-born varies from 4.6% in Hungary and 9.5% in Italy to more than 10% in the Netherlands (11.5%), the UK (12.6%), Spain (13.2%) and Germany (13.6%) [22, 23]. The UK, the Netherlands and Spain have been described as having a ‘difference-based’ or communitarian approach to migration and diversity, by recognising difference, actively adapting services to diversity and providing tailored (as opposed to mainstream) health care services [24]. The ‘difference-blind’ or ‘republication’ systems of Germany, Italy and Hungary assume all citizens should be treated equally through the provision of mainstream (as opposed to separatist) services that are passive to diversity and operate with expectations of assimilation by migrants [25]. The six study countries also vary in regard to the financing available for health care. For instance, Germany and the Netherlands spend around five times the amount (per capita) on health care than Hungary (4753.9 USD and 5456.5 USD (respectively) vs. 991.3 USD in 2012) [26].

It is likely that these differences in experience of migration, of socio-cultural value system response to diversity and in health care financing will affect the availability of language support services in the six study countries. In this exploratory study, we investigated the availability of language support services (interpreters and translated written materials about the virus/disease) for linguistic minority chronic viral hepatitis patients. We also investigated health care professionals’ agreement that language barriers are explanations of the lack of hepatitis B/C screening among people born in medium/high prevalence countries in primary care, the low uptake of screening among these patients and why people diagnosed with a chronic infection do not reach specialist care. This study is part of HEPscreen ([http://www.hepscreen.eu]), an EU Health Programme-funded project generally focused on screening for chronic viral hepatitis among migrants in Europe and specifically focused on these six countries.

## Methods

We developed an online survey and sent it to a large convenience sample (*n* = 1026) of expert clinicians involved in screening or care for viral hepatitis in six EU countries: Germany, Hungary, Italy, the Netherlands, Spain and the UK (England, Wales and Scotland). Recipients were identified via a comprehensive snowballing method via stakeholder consultation within our HEPscreen project consortium, and membership of professional networks or clinical associations involved in five areas of health care: general practice/family medicine, antenatal care, health care for asylum seekers, sexual health services, and specialist secondary care. The aim was to reach knowledgeable experts able to reflect on the circumstances in their profession and country rather than use a representative sampling framework among individual clinicians. We took a health system approach, looking across countries rather than within specific health care services.

The survey measured availability of written and oral language support services (translated materials and telephone or face-to-face interpreters) using closed questions and a three-point ordinal scale: ‘very common’, ‘variable or not routinely’ and ‘rarely or never’ (unsure was also available). We also measured clinicians’ perception, using three closed questions and a five-point Likert scale of agreement, of how far language barriers explain three scenarios: the low uptake of screening for hepatitis B/C among migrants born in medium/high prevalence countries; the lack of screening by primary care services among migrants with country of birth-related risk factors; and why people diagnosed with a chronic infection do not reach specialist care for further investigation and antiviral treatment. These three scenarios respectively reflect patient-related, health care service-related and health care system-related issues. The questions reported here are specific sections of a larger survey aimed at understanding screening, referral, treatment and clinical management of hepatitis B/C patients in the six countries. Findings from other sections will be and are reported elsewhere [27].

The survey was pre-tested five times in English in three of the study countries, each with a professional from each of the five professions/health care sectors: from general practice (in Italy), an antenatal care (in Germany), health care for asylum seekers (in Italy), sexual health (in the Netherlands), and specialists in hepatology/gastroenterology (in the Netherlands)). The method used to pre-test was influenced by cognitive interviewing techniques which allow for every detail, no matter how trivial, to be captured by asking subjects to ‘think out loud’ about the question and answer options. Interviewers can gauge how well the subject has interpreted and understood each aspect of the survey. These techniques also allow for ambiguous or unfamiliar terms and questions to be identified [28]. Feedback from each interview was discussed within the research team and a consensus was reached on each proposed amendment or addition. Several minor changes were made to each survey following pre-testing. Please see the Additional files 1, 2, 3, 4 and 5 available online for the final version of the survey.

A professional translation company was used to translate the survey into the languages of the study countries. To ensure the versions provided were understandable, accurate and professional, a native speaker (and fluent English speaker) from the HEPscreen project consortium checked each language translation.

The survey was sent via email in July 2012. Two further reminders were sent and the survey finally closed in September 2012. The reminder schedule conformed to the deadlines set out within our EU Health Programme milestone framework (achievement of which was a condition of our funding). Data were anonymised, extracted and a descriptive analysis was performed using SPSS 19.02. We calculated proportions at the country level for both questions about language support availability and about agreement with language barriers as explanations. To account for different numbers of respondents across each of the five health services, we calculated a weighted average by summing the proportions in each response category in each survey and dividing by five.

## Results

We received a total of 238 responses from 1026 recipients (23% response rate). The distribution across the six countries was: 17 in Spain, 21 in Hungary, 42 in the UK, 49 in the Netherlands, 52 in Italy and 57 in Germany. The total included representatives from each of the five areas of health care in all six countries, 81% of whom have a clinical role/are involved in the care of patients. The health care professions/areas of expertise of the 238 respondents were as follows: 87 (37%) from antenatal care, 64 (27%) specialists in gastroenterology/hepatology or infectious diseases (in secondary care), 40 (17%) from general practice/family medicine, 29 (12%) from sexual health/genito-urinary medicine, and 18 (8%) from health care for asylum seekers and refugees.

### Availability of language support

Of all six study countries, translated materials in languages other than the national language were most commonly available in the Netherlands and Germany where just over one third (35 and 37%) indicated very common. However, a large proportion (44 and 36% respectively) indicated they were variably available (Table 1). Translated materials were least commonly available in Italy, where 80% of respondents indicated ‘rarely or never’ along with the majority in Hungary (61%) and Spain (60%). Half in the UK (51%) indicated translated materials were variably or not routinely available, with the other half of respondents distributed in all of the other response categories. Interpreters are also very commonly available in the Netherlands (60%), and in the UK, where over half (54%) indicated very common and no-one indicated rarely or never (also Table 1). In contrast, interpreters are rarely or never available for over half in Italy (56%) and nearly half (45%) in Germany, along with over a third in Hungary. Interpreters in Spain seem to be more common than translated materials, which is a general trend seen in our data except for in Germany where translated materials appear to be more commonly available.

**Table 1:** Availability of translated materials (TM) and interpreters (I) in the six countries

	DE (*n* = 57)		HU (*n* = 21)		IT (*n* = 52)		NL (*n* = 49)		ES (*n* = 17)		UK (*n* = 42)	
	TM	I	TM	I	TM	I	TM	I	TM	I	TM	I
Very common	35%	10%	6%	14%	5%	2%	37%	60%	15%	25%	20%	54%
Variable or not routinely	36%	23%	16%	40%	13%	38%	44%	24%	20%	50%	51%	36%
Rarely or never	17%	45%	61%	36%	80%	56%	14%	10%	60%	23%	13%	0%
Unsure	12%	22%	17%	11%	2%	4%	6%	6%	5%	3%	17%	11%

*Abbreviations*: *DE* Germany, *HU* Hungary, *IT* Italy, *NL* the Netherlands, *ES* Spain, *UK* United Kingdom

### Language barriers as explanations

In the UK, over half agree or strongly agree that language barriers explain all three scenarios (screening uptake, screening offer and referral), and only a minority (between 7 and 15%) expressed disagreement (Table 2). Strongest agreement in the UK emerges about the role of language barriers in referral, where nearly three quarters (73%) strongly agree that these explain why cases do not reach secondary care. A similar pattern emerges in Germany, where three quarters of respondents agree/strongly agree that language barriers are explanations. Most agreement (over 75%) in Germany is seen for the notion of language barriers as explanations of the lack of screening by primary care services. A less conclusive pattern is found in the Netherlands, where, although between 40 and 55% agree that language barriers are explanations of all three scenarios, a large proportion are neutral and a significant minority disagree/strongly disagree that language barriers explain the low uptake of screening (33%) and why infected patients do not reach secondary care (19%). An interestingly divergent pattern is seen in Hungary, especially in response to language barriers as explanations of low uptake and of why cases do not reach secondary care. Whilst nearly half in Hungary disagree/strongly disagree that language barriers explain the low uptake of screening, over a third agree/strongly agree that they do explain the low screening uptake. Similarly, nearly half (44%) agree/strongly agree that language barriers explain why cases of chronic viral hepatitis do not reach specialist care, one third in Hungary disagree/strongly disagree with this notion. There is agreement (77%) that language barriers explain why screening is not offered by primary care services in Hungary. A similarly divergent view is seen in Italy; 80% agree that language barriers explain the lack of screening in primary care, no such strong consensus emerges regarding the other two issues. Although around half agree/strongly agree that language barriers explain a low screening uptake and why cases do not reach secondary care, a significant minority disagree, especially so about the lack of screening offer (26%). In Spain, there is also some diversity in opinion although around two thirds (68%) agree that language barriers are explanations of the lack of screening in primary care and why hepatitis B/C cases do not reach secondary care. Variety in perception of language barriers as explanations of low screening uptake is seen, with 40% in agreement/strong agreement and 33% in disagreement/strong disagreement.

**Table 2:** Scale of agreement that language barriers explain three scenarios

Scenario	Response option	DE (*n* = 31)	HU (*n* = 18)	IT (*n* = 35)	NL (*n* = 49)	ES (*n* = 15)	UK (*n* = 39)
Language barriers explain the low uptake of screening by people with country of birth-related risk factors	Strongly disagree	3%	18%	0%	2%	13%	3%
	Disagree	10%	35%	26%	31%	20%	13%
	Neutral	23%	12%	26%	22%	27%	23%
	Agree	52%	24%	31%	39%	33%	54%
	Strongly Agree	13%	12%	17%	6%	7%	8%
A lack of translated materials/interpreters explains the lack of screening in primary care	Strongly disagree	0%	6%	0%	0%	0%	0%
	Disagree	7%	6%	6%	14%	13%	15%
	Neutral	16%	12%	14%	45%	27%	33%
	Agree	68%	59%	63%	37%	53%	44%
	Strongly Agree	10%	18%	17%	4%	7%	8%
Language barriers explain why hepatitis B/C cases do not reach specialist secondary care	Strongly disagree	4%	22%	0%	0%	0%	0%
	Disagree	13%	11%	15%	19%	18%	7%
	Neutral	17%	22%	35%	26%	18%	20%
	Agree	58%	33%	39%	41%	46%	63%
	Strongly Agree	8%	11%	12%	15%	18%	10%

*Abbreviations*: *DE* Germany, *HU* Hungary, *IT* Italy, *NL* the Netherlands, *ES* Spain, *UK* United Kingdom

## Discussion

European countries have differing historical experiences of migration, with the six countries in our study illustrative both of these differences and of the availability services to overcome barriers to health care, in this instance language barriers. Our first aim was to understand availability of language support (translated materials and interpreters) in health care services most involved in screening and/or treating chronic hepatitis B/C among at risk migrant communities. Results suggest that translated materials are rarely or never available in Hungary, Italy and Spain but more commonly or variably available in the Netherlands, Germany and the UK. Our results suggest that interpreters are quite commonly available in the UK, the Netherlands and Spain but more variably or rarely available in Germany, Hungary and Italy. Our second research aim was to investigate how far professionals agree that language barriers explain three scenarios: the low uptake of screening among people with country of birth-related risk factors; the lack of screening in primary care among these risk groups; and why cases of chronic viral hepatitis do not reach specialist care for clinical management and treatment. Three interesting results emerge from this second research question: one, that opinion about the role of language barriers in the three scenarios is not identical in each country; two, that differences of opinion within one country about each scenario exist; and three, that professionals in countries with the most infrequent availability (Hungary and Italy) disagree most that language barriers are explanations.

Our findings both mirror and contrast with those from other studies about language support in health services in these six study countries and about barriers to screening and referral for chronic viral hepatitis internationally. In an analysis of migrant health policies, the provision of interpreters was found to be detailed in policy goals in the UK, the Netherlands and Spain although actual implementation of policy was considered patchy [26], which is what we see in our results for these three countries. A summary study in Germany similarly found that the use of interpreters in health care is not well-established and that availability is the exception not the rule [29], again in line with the variable or not routine availability reported by 36% of respondents here. Other studies from the Netherlands seem to suggest a less frequent availability of support services than we see reported in our study; studies found poor information exchange between migrant patients and health care professionals, an underreporting of poor Dutch proficiency in medical records, that family members are used as interpreters and that professional interpreter services are hardly used in hospital settings [30, 31].

In the UK, the Netherlands and Germany, migration from the former Empire (in the case of the UK) and from the Mediterranean region (in the Netherlands and Germany) has been an historic trend since the 1950s, although war, conflict and economic crisis in the Eastern Mediterranean region has led to an influx of migrants presenting new challenges to health care systems. In contrast, migration to Spain and Italy is a relatively new phenomenon and it is only in the last two decades that these countries have experienced migration in large numbers from, for example, 2.5% in 2001 in Spain to 13.2% in 2012 [32]. Migration to Hungary is still relatively uncommon with just 4.6% of the population foreign-born in 2012 [23]. Given these differences in the population of migrants, disagreement about the role that language barriers play in screening and referral could be explained by how likely it is that professionals encounter linguistic minority patients in their services. Disagreement about their role a could reflect a perception that people with country of birth-related risk factors are not linguistic minorities (due to speaking the same/similar languages) and/or can speak the national language to a good enough standard not to require support. The results could also reflect underlying socio-cultural value systems that migrants should assimilate and adapt by learning the national language. Indeed, interpreters are reported to be less common in the three ‘difference-blind’ systems in our study, namely Germany (10%), Hungary (14%) and Italy (2%) compared to the ‘difference-based’ systems of the UK (54%), the Netherlands (60%) and Spain (25%). Agreement that language barriers exist is however most strong in Germany, suggesting that clinicians may not subscribe to the socio-cultural value system of assimilation over adaptation. Agreement in the Netherlands is surprisingly low across the three scenarios, which could be explained either by the common availability of interpreters we see in the results from our first aim (and therefore removal of language barriers) or by the socio-political shift from multi-culturialist ‘difference-based’ policies to inter-culturalist policies that favour individual responsibility and encourage migrants to learn Dutch [24]. A recent study from the Netherlands offers some support for this notion and found that whilst it is hospital policy to make (hospital-funded) interpreters available, nursing service heads rarely reference the policy, and health care providers indicated that it is the responsibility of patients to overcome language barriers by bringing an interpreter to appointments [30]. Similarly, public funding for interpreters in health care was recently withdrawn [33]. Disagreement about the role of language barriers in the three scenarios could also reflect a perception of prioritisation i.e. that language barriers are not as important explanations when compared to other factors such as health care provider knowledge, awareness of country of birth as a risk factor, or a lack of time in health care appointments [34]. A study from the US found that hepatitis C testing is rarely performed in primary care among patients presenting with infection risk factors, although the list in the study did not include birth in an endemic country, itself an indication of the lack of awareness about this important risk group [35]. Another study in the US found frequent reports of communication barriers between physicians and CHC patients, including stigmatisation, assumptions of sexual promiscuity or injecting drug use as the source of infection, a lack of disease-related explanation or post-test counselling, and an unwillingness to refer [36]. The finding that stigmatisation and assumptions are made about patients infected with viral hepatitis, about people with the additional barrier of language, increases the likelihood of these patients receiving poor quality health care. A study in Australia about barriers faced by migrants in accessing health care for viral hepatitis infection found that language barriers was the ‘chief barrier’ for 45% of patients with a migrant background [8]. Studies from the UK, the Netherlands and Italy show that a large proportion of chronic viral hepatitis patients do not reach secondary care [6, 9, 37, 38] and that immigration is negatively associated with being on treatment [39, 40]. These studies suggest multiple explanations for why screening isn’t offered to or taken up by at-risk migrant groups as well as why diagnosed patients do not reach specialist care. However, to realise the public health gains possible due to improved treatment regimens, screening and referral needs to be scaled up [41].

A strength of this study is the inclusion of study countries that reflect different models and value systems in health care delivery. Previous multi-country research among expert clinicians and policy makers about the provision of hepatitis B/C screening and treatment services for at risk populations has only been conducted in English [42, 43]. The translation of our survey into the national languages of our study countries is a concerted effort to overcome language barriers, an important strength given the focus of the study on language barriers themselves. A further strength is the inclusion of experts across the patient pathway, from primary to secondary care as well as specific services for refugees and asylum seekers. It is notoriously challenging to yield high response rates to non-incentivised online surveys among busy, practising clinicians. We reached 238 knowledgeable experts in five areas of health care in the six countries and, although the overall response rate is low (23%), the results in all six countries are broadly in line with the scarce disease-specific, migrant population-specific and European-focused research available to compare and contrast our findings with.

## Conclusions

Our findings show pronounced differences between countries in the availability of interpreters, differences that mirror the underlying socio-cultural value systems of ‘difference-sensitive’ and ‘difference-blindness’ that have been described in literature. Results also suggest varying or service-/professional-specific availability of interpreters and/or translated written materials for chronic viral hepatitis. This is despite the complexity of the disease, the recognised barriers to screening and care, and the large undiagnosed burden among (potentially) linguistic minority migrant groups. This finding is mirrored in the view among many clinicians in the six study countries that language barriers are important explanations of low screening uptake, a lack of screening by primary care, and why diagnosed hepatitis B/C patients do not reach secondary care. Europe is behind the curve of viral hepatitis-related mortality and getting ahead requires expanding and improving access to screening among at risk populations, especially among people with country of birth-related risk factors. Evidence shows that interpreters and translated materials can improve acceptance of screening, patient knowledge and understanding, and, most importantly, clinical outcomes. To overcome language barriers, it is important that existing and future screening programmes provide language support for linguistic minority patients at risk of or diagnosed with chronic viral hepatitis.

## Additional files

Additional file 1: Antenatal screening survey. (PDF 309 kb)
Additional file 2: Asylum seeker/refugee survey. (PDF 324 kb)
Additional file 3: General Practitioner survey. (PDF 353 kb)
Additional file 4: Screening for viral hepatitis in Sexual Health Services/by Genit-Urinary Medicine specialist clinics survey. (PDF 344 kb)
Additional file 5: Specialist secondary care survey. (PDF 359 kb)

## Abbreviations

CHB: Chronic hepatitis B
CHC: Chronic hepatitis C
DAAs: Direct acting antivirals
DE: Germany
ES: Spain
EU: the European Union
HU: Hungary
I: Interpreters
IT: Italy
NL: The Netherlands
TM: Translated materials
UK: The United Kingdom
US: United States of America
USD: United States Dollars
