# Supplementary material for: Language support for linguistic minority chronic hepatitis B/C patients: an exploratory study of availability and clinicians’ perceptions of language barriers in six European countries
Source: BMC Health Serv Res. 2017 Feb 20;17:150. doi: 10.1186/s12913-017-2095-5 (PMC5319068; doi:10.1186/s12913-017-2095-5)
Supplement: Additional file 1: — Antenatal screening survey. (PDF 309 kb) [file 12913_2017_2095_MOESM1_ESM.pdf]

Antenatal screening

Welcome text

You have been approached to complete this survey as an expert in your professional field of antenatal care, and/or as part of your involvement in a national or regional level organisation that represents clinicians and/or public health professionals. It is from this representative position that we would like you to respond to the survey. We are interested in the general or routine viral hepatitis related screening and clinical management practices in your country.

This survey has been sent to experts in seven different countries in the EU. We recognise that practices will differ between the seven countries and have tried to reflect this diversity in the structure and answering options. Although there may be aspects of the survey that are not applicable to the situation in your country, the structure of the survey will enable you to reflect that in your answers.

The survey will take approximately 15-20 minutes. It is possible to stop and complete the survey at a later time.

Please be assured that your responses will be kept anonymous.

Section 1: Respondent profile

These questions relate to your involvement in a national or regional level organisation that represents clinicians and/or public health professionals and/or patients.

1. What is the name of your organisation? ……………..

2. What type of organisation is it?
    - NGO
    - National Government
    - Regional Government
    - Clinical Association
    - Professional Organisation
    - University
    - Patient association
    - Other (please specify): ……….

3. What is your Job Role/Job Title: ……….

4. Do you also have clinical responsibilities and are directly involved in the care of patients?
    - Yes
    - No [skip to section 2]

5. What type of medical facility do you work in?
    - GP practice
    - Public health service/health protection unit
    - Clinic (outside a hospital)
    - General hospital
    - University/Teaching hospital
    - Health care service at receiving center/national border control

6. What is your medical specialism/clinical role?
   - General Practitioner
   - Infectious Disease specialist
   - Gastroenterologist/Hepatologist
   - Gynaecologist/Obstetrician
   - Other (please specify): …………

7. How often do you see patients with a chronic hepatitis B or hepatitis C infection?
   - Never
   - A few patients per year (1-10)
   - On a monthly basis
   - On a weekly basis

Section 2: Hepatitis B screening/testing practices:

8. In your experience what is the **standard antenatal screening/testing practice for hepatitis B** in your country? Is hepatitis B screening/testing offered to pregnant women?
   - Yes – to all on a regular basis
   - Yes – but only sporadically
   - No
   - Unsure
9. [If YES to screening/testing of pregnant women]
   Is co-payment/contribution required from pregnant women for antenatal hepatitis B screening/testing?
   - No - free for all
   - Yes - contribution required from all
   - Only free for some (please indicate which women)
   - Unsure
10. [If YES to screening/testing of pregnant women]
    After screening, are hepatitis B negative women **vaccinated** (i.e. those not already vaccinated)?
    - Yes – post birth by the antenatal care provider
    - Yes – post birth by another health care service provider
    - Seldom – only under special circumstances
    - No – generally not
    - Unsure
11. [If Yes or Seldom to vaccination of pregnant women]
    Is **individual co-payment/contribution** required from women for hepatitis B **vaccination**?
    - No - free for all
    - Yes - contribution required from all
    - Only free for some (please indicate which women)
    - Unsure
12. In your experience, do antenatal hepatitis B screening/testing practices differ between administrative regions in your country?
    - Yes – screening practices differ between regions
    - No – screening practices are the same across the country
    - Unsure

Section 2: Hepatitis C screening/testing practices:

13. In your experience what is the **standard screening/testing practice for hepatitis C** in your country? Is screening/testing for hepatitis C offered to pregnant women?
    - Yes – to all on a regular basis
    - Yes – but only sporadically
    - No
    - Unsure
14. [If YES to screening/testing of pregnant women]
    Is **individual co-payment/contribution** required from pregnant women for antenatal hepatitis C screening/testing?
    - No – free for all
    - Yes – contribution required from all
    - Only free for some (please indicate which subgroups)
    - Unsure
15. In your experience, do antenatal hepatitis C screening/testing practices differ between administrative regions in your country?
    - Yes – screening practices differ between regions
    - No – screening practices are the same across the country
    - Unsure

Section 3: Pre-test information and advice (counselling) before testing

16. Who provides pregnant women with information and advice (counselling) before a test for viral hepatitis?
    - ☐ Pre-test information is not provided
    - ☐ Midwives
    - ☐ Medical assistants
    - ☐ Obstetrician/Gynaecologist
    - ☐ General Practitioners
    - ☐ Public health services/health protection units
    - ☐ Infectious Disease specialists (not located in public health services/health protection units.)
    - ☐ Others
17. Content of pre-test information
    How common is it to provide information and advice to pregnant women on the following hepatitis B related topics before screening/testing for hepatitis B:

	Very common	Variable or not routinely	Rarely or never	Unsure
The need to vaccinate the baby post birth				
General information about the virus				
The test itself				
Implications of a positive test				
Future effect on relationships/social network				
Support available to patient				
Treatment options				

Section 4: Part 1: Disease-related advice and guidance (counselling) following a positive result:

18. **Who informs** pregnant women of a positive result?
    - Midwives
    - General Practitioners
    - Public health services/health protection units
    - Infectious disease specialists specialists (not in public health services/health protection units)
    - Specialists (e.g. Gastroenterologists/Hepatologists)
    - Obstetricians/Gynaecologists
    - Other
    - Unsure
19. **Who has the main responsibility** for providing disease-related advice and guidance (**counselling**) to pregnant women following a positive diagnosis of viral hepatitis in your country?
    - Midwives/maternity units
    - Obstetricians/Gynaecologists
    - General Practitioners
    - Public health services/health protection units
    - Infectious disease specialists specialists (not in public health services/health protection units)
    - Specialists (e.g. Gastroenterologists/Hepatologists)
    - Other

20. [If YES to hepatitis B screening/testing offered to pregnant women]
    Are **midwives/antenatal care providers** involved in the care of hepatitis B positive pregnant women for any of the following:

	Yes	No	Unsure
Disease-related advice and guidance (counselling)			
Screening/testing for other hepatitis viruses			
Contact tracing			
Patient management			

21. [If YES to hepatitis C screening/testing offered to pregnant women]
    Are midwives/antenatal care providers involved in the care of hepatitis C positive pregnant women for any of the following:

	Yes	No	Unsure
Disease-related advice and guidance (counselling)			
Screening/testing for other hepatitis viruses			
Contact tracing			
Patient management			

22. Are pregnant women **referred to other health services/professionals** for disease-related advice and guidance (**counselling**) following a positive diagnosis for viral hepatitis?
    - Yes - mostly pre birth
    - Yes - mostly post birth
    - No
    - Unsure

23. [If YES to referring pregnant women for hepatitis C counselling]

Which health services/professionals are they **referred to**?

- ☐ Public health services/health protection units
- ☐ Infectious Disease specialists (not in public health services/health protection units)
- ☐ Gastroenterologists/Hepatologists
- ☐ Obstetrician/Gynaecologists
- ☐ General Practitioner
- ☐ Other:

Section 4: Part 2: Content of disease-related advice and guidance (counselling):

24. How common is it to include the following topics in **disease-related advice and guidance (counselling)** to hepatitis B positive **pregnant women:**

	Very common	Variable or not routinely	Rarely or Never	Unsure
General information about the disease				
Hygiene measures to protect transmission to others				
Contact tracing				
Other tests required				
What to expect, onward referral				
Treatment options, benefits and side effects				
The importance of a healthy lifestyle, especially the damaging role of alcohol consumption				
Mental health promotion and staying positive				
Patient organisations / support groups				
The need to vaccinate the baby post birth				
Breastfeeding				
Implications for delivery				

Section 5: Screening/testing positive patients for other hepatitis viruses:

25. [If YES to hepatitis C screening/testing offered to pregnant women]
Are hepatitis B positive women screened/tested for hepatitis C:

- o Yes - pre birth
- o Yes - post birth
- o No
- o Unsure

Section 6: Referral and clinical management:

26. Which hepatitis B positive women are referred to onward care for chronic viral hepatitis?

- o All women
- o A selection based on clinical indicators
- o None

27. [If YES to hepatitis C screening/testing offered to pregnant women]

- o All women

- ○ A selection based on clinical indicators
- ○ None

28. [If answered a selection based on clinical indicators to question 27]
What are these clinical indicators?
- ☐ Viral load
- ☐ HBe antigen status
- ☐ ALT
- ☐ Unsure
- ☐ Other:

29. Who can midwives/maternity services refer chronic viral hepatitis cases to?
- ☐ Unsure
- ☐ Directly to the Gynaecologist
- ☐ Directly to specialist secondary care
- ☐ Referral to specialist secondary care via GP
- ☐ Referral to specialist secondary care is via another service (please give details)

30. [If answered referral is via another service]
If referral to specialist secondary care is via another service, please give details here____

<u>Section 7: Contact tracing and vaccination:</u>

31. Is **hepatitis B screening** offered to **household and/or sexual contacts** of hepatitis B positive women?
- ○ No
- ○ Yes – All contacts
- ○ Yes – a selection of contacts (please specify)
- ○ Unsure

32. [If <u>YES</u> to hepatitis B screening/testing offered to household and/or sexual contacts of hepatitis B positive women]
Are hepatitis B negative contacts (**household and/or sexual contacts**) of hepatitis B positive women vaccinated?
- ○ No
- ○ Yes – All hepatitis B negative contacts
- ○ Yes – a selection of hepatitis B negative contacts (please specify)
- ○ Unsure

33. [If <u>YES</u> to hepatitis B screening/testing offered to household and/or sexual contacts of hepatitis B positive women]
Who has the main responsibility for the screening/testing of contacts for hepatitis B?
- ○ Public health services/health protection units
- ○ Infectious disease specialists (not in public health services/health protection units)
- ○ Gastroenterologists/Hepatologists
- ○ Obstetrician/Gynaecologists
- ○ General Practitioner
- ○ Other

34. [If YES to vaccination of hepatitis B negative contacts of hepatitis B positive women]
Who has the main responsibility for the vaccination of contacts?
- Public health services/health protection units
- Infectious disease specialists (not in public health services/health protection units)
- Gastroenterologists/Hepatologists
- Obstetrician/Gynaecologists
- General Practitioner
- Other

Section 8: Professional practice and training:

35. How common is it for midwives/antenatal care providers to have the following available:
Please choose the appropriate response for each item:

	Very common	Variable or not routinely	Rarely or never	Unsure
Materials about viral hepatitis in the national language				
Materials about viral hepatitis in other languages				
Interpreter services via a telephone				
Face to face interpreter services				

36. Is training available for antenatal care providers to improve knowledge and skills about viral hepatitis?
- Yes (please give details)
- No
- Unsure

Section 8: Professional practice and training for Hepatitis B:

37. Are there any official national guidelines about **hepatitis B** screening and patient management in place in your country? If yes, please give name and publisher.

Guidelines	
☐ General Hepatitis B guidelines	
☐ Specific guidelines for antenatal services or pregnant women	
☐ Other hepatitis B guidelines	

38. [If YES to official national guidelines about Hepatitis B screening and patient management in place in your country]
Does this specific guideline for antenatal services or pregnant women include information about:
- ☐ Pre-test information and advice for patients
- ☐ Disease-related advice and guidance (counselling) for patients following positive diagnosis
- ☐ Onward referral of chronic viral hepatitis patients to other services
- ☐ Recommendations to tailor services or information for people from a migrant or ethnic minority background
- ☐ Assessment of liver disease
- ☐ Treatment strategies

Section 8: Professional practice and training for Hepatitis C:

39. Are there any official national guidelines about **hepatitis C** screening and patient management in place in your country? If yes, please give name and publisher.

Guidelines	
☐ General Hepatitis C guidelines	
☐ Specific guidelines for antenatal services or pregnant women	
☐ Other hepatitis C guidelines	

40. [If YES to official national guidelines about Hepatitis C screening and patient management in place in your country]
Does this specific guideline for antenatal services or pregnant women include information about:
- ☐ Pre-test information and advice for patients
- ☐ Disease-related advice and guidance (counselling) for patients following positive diagnosis
- ☐ Onward referral of chronic viral hepatitis patients to other services
- ☐ Recommendations to tailor services or information for people from a migrant or ethnic minority background
- ☐ Assessment of liver disease
- ☐ Treatment strategies

Section 9: Barrier questions:

Disease-related counselling, onward referral and clinical management of hepatitis B/C positive pregnant women.

41. To what extent do you agree with the following statements as explanations of why pregnant women infected with chronic hepatitis B/C do not reach specialized health care (e.g. hepatologists) for further investigation and treatment in your country.

**Statements**	**Strongly disagree**	**Disagree**	**Neither agree nor disagree**	**Agree**	**Strongly agree**
There is confusion among health professionals about which services are involved at what stage of care for pregnant women infected with chronic hepatitis and hence patients get lost in the referral process					
The focus is mainly on vaccination of the baby, not on treatment of the women testing positive					
Other than the consequences for the baby (such as vaccination), newly diagnosed pregnant women generally do not receive counselling on the consequences of the disease.					
There are few specialists to whom pregnant women can be referred to for specialized care					
Referral to the specialist is postponed until after birth					
The antiviral treatment itself is generally not covered under the general health care service/insurance scheme in my country					

Patients are referred to the specialist but refuse further investigation or treatment					
There is limited guidance available to primary health care professionals about onward referral, counselling and patient management of hepatitis B/C patients					
Although training on viral hepatitis management is available to antenatal care providers, uptake is generally low among professionals.					
Women from a migrant or ethnic minority background face language barriers when visiting health services (limited availability of translated materials or interpreter services)					

42. If you think there are other explanations, please give details in the box below.

Comments

43. If you have any comments in general or regarding the screening, counseling, referral and/or treatment of Hepatitis B/C in your country, we would be grateful to learn more and benefit from your experience. Please write any remarks in the text box below.

Thank you for completing this survey.
