# Supplementary material for: Language support for linguistic minority chronic hepatitis B/C patients: an exploratory study of availability and clinicians’ perceptions of language barriers in six European countries
Source: BMC Health Serv Res. 2017 Feb 20;17:150. doi: 10.1186/s12913-017-2095-5 (PMC5319068; doi:10.1186/s12913-017-2095-5)
Supplement: Additional file 2: — Asylum seeker/refugee survey. (PDF 324 kb) [file 12913_2017_2095_MOESM2_ESM.pdf]

Asylum seekers

Welcome text

You have been approached to complete this survey as an expert in your professional field of antenatal care, and/or as part of your involvement in a national or regional level organisation that represents clinicians and/or public health professionals. It is from this representative position that we would like you to respond to the survey. We are interested in the general or routine viral hepatitis related screening and clinical management practices in your country.

This survey has been sent to experts in seven different countries in the EU. We recognise that practices will differ between the seven countries and have tried to reflect this diversity in the structure and answering options. Although there may be aspects of the survey that are not applicable to the situation in your country, the structure of the survey will enable you to reflect that in your answers.

The survey will take approximately 15-20 minutes. It is possible to stop and complete the survey at a later time.

Please be assured that your responses will be kept anonymous.

Section 1: Respondent profile

These questions relate to your involvement in a national or regional level organisation that represents clinicians and/or public health professionals and/or patients.

1. What is the name of your organisation? ……………..

2. What type of organisation is it?
    - NGO
    - National Government
    - Regional Government
    - Clinical Association
    - Professional Organisation
    - University
    - Patient association
    - Other (please specify): ……….

3. What is your Job Role/Job Title: ……….

4. Do you also have clinical responsibilities and are directly involved in the care of patients?
    - Yes
    - No [skip to section 2]

5. What type of medical facility do you work in?
    - GP practice
    - Public health service/health protection unit
    - Clinic (outside a hospital)
    - General hospital
    - University/Teaching hospital
    - Health care service at receiving center/national border control

6. What is your medical specialism/clinical role?
   - General Practitioner
   - Infectious Disease specialist
   - Gastroenterologist/Hepatologist
   - Gynaecologist/Obstetrician
   - Other (please specify): …………

7. How often do you see patients with a chronic hepatitis B or hepatitis C infection?
   - Never
   - A few patients per year (1-10)
   - On a monthly basis
   - On a weekly basis

Section 2: Hepatitis B screening/testing practices:

8. In your experience what is the standard **hepatitis B screening/testing practice for asylum seekers** in your country? Is hepatitis B screening carried out among asylum seekers?
   - No
   - Yes – on a regular basis
   - Only in cases where indicated (please specify)
   - Unsure
9. [If YES to hepatitis B screening carried out among asylum seekers]
   Are hepatitis B **negative individuals vaccinated**?
   - No
   - Yes – all
   - Yes – a selection (please indicate which people)
   - Unsure
10. [If YES to vaccination of hepatitis B negative individuals]
    Is **individual co-payment/contribution** required?
    - Yes – contribution required from all
    - No – free for all
    - Only free for some (please indicate which people)
    - Unsure
11. In your experience, do asylum seeker related hepatitis B screening/testing practices differ between administrative regions in your country?
    - Yes – screening practices differ between regions
    - No – screening practices are the same across the country
    - Unsure

Section 2: **Hepatitis C** Screening /testing practices:

12. In your experience what is the standard hepatitis C **screening/testing practice for asylum seekers** in your country? Is hepatitis C screening carried out among asylum seekers?
    - No
    - Yes – on a regular basis
    - Only in cases where indicated (please specify)
    - Unsure
13. In your experience, do asylum seeker related hepatitis C screening/testing practices differ between administrative regions in your country?
    - Yes – screening practices differ between regions
    - No – screening practices are the same across the country
    - Unsure

Section 3: **Pre-test** information and advice (counselling) before testing:

14. Who provides asylum seekers with information and advice **before a test** for viral hepatitis?

- ☐ Pre-test information is not provided
- ☐ Health care service at receiving centres/national border control
- ☐ General Practitioners
- ☐ Public health services/health protection units
- ☐ Infectious disease specialists (not located in health care service at receiving centre/national border control)
- ☐ Voluntary organizations
- ☐ Unsure
- ☐ Others (please specify)

Content of pre-test information

15. [If Pre-test information is provided]
How common is it to provide information and advice (counselling) to asylum seekers on the following topics before screening/testing for hepatitis B or hepatitis C:

	Very common	Variable or not routinely	Rarely or never	Unsure
Modes of transmission				
General information about the virus				
The test itself				
Implications of a positive test				
Access to health care				
Future effect on relationships / social network				
Support available to patient				
Treatment options				

Section 4: Part 1: Desease-related advice and guidance (counselling) following a positive result:

16. Who informs the patient of a positive result?

- o Health care service at receiving centres/national border control
- o General Practitioners
- o Public health services/health protection units
- o Infectious disease specialists (not in public health services/health protection units)
- o Specialists (e.g. Gastroenterologists/Hepatologists)
- o Other (please specify)
- o Unsure

17. Who has the **main responsibility** for providing **disease-related counselling/advice and guidance** to asylum seeker patients following a positive diagnosis for viral hepatitis?

- o Health care service at receiving centres/national border control
- o General Practitioners
- o Public health services/health protection units
- o Infectious disease specialists (not in public health services/health protection units)
- o Specialists (e.g. Gastroenterologists/Hepatologists)
- o Other (please specify)
- o Unsure

Section 4: Part 2: Content of disease-related advice and guidance (counselling) following a positive result:

18. How common is it to include the following topics in disease-related advice and guidance to asylum seeker following a positive test result for hepatitis B and/or C:

	Very common	Variable or not routinely	Rarely or never	Unsure
General information about the disease				
Hygiene measures to protect transmission to others				
Contact tracing				
Other tests required				
What to expect, onward referral				
Treatment options, benefits and side effects				
The importance of a healthy lifestyle, especially the damaging role of alcohol consumption				
Mental health promotion and staying positive				
Patient organisations / support groups				
Access to health care				

Section 5: Screening/testing positive patients for other hepatitis viruses:

19. Are **hepatitis B positive patients** screened/tested for hepatitis C?

○ No
○ Yes – all patients
○ Yes – a selection of patients (please specify which)
○ Unsure

20. Are **hepatitis C positive patients** screened/tested for hepatitis B?

○ No
○ Yes – all patients
○ Yes – a selection of patients (please specify which)
○ Unsure

21. [If YES to screening hepatitis B positive patients for hepatitis C or YES to screening hepatitis C positive patients for hepatitis B]

Who has the main responsibility for screening/testing of hepatitis B or hepatitis C positive patients for other hepatitis viruses?

○ Health care service at receiving centres/national border control
○ General Practicitioners
○ Public health services/health protection units
○ Hospitals/clinics
○ Sexual Health Services
○ Other (please specify)

22. Are **hepatitis C positive patients** who are found to be hepatitis B negative vaccinated against hepatitis B?

○ No
○ Yes – all patients
○ Yes – a selection of patients (please specify which)
○ Unsure

23. [If YES to vaccination of hepatitis C positive patients who are found to be hepatitis B negative vaccinated against hepatitis B]

Who has the main responsibility for vaccination of hepatitis C positive patients found hepatitis B negative?

- ○ Health care service at receiving centres/national border control
- ○ General Practicitioners
- ○ Public health services/health protection units
- ○ Hospitals/clinics
- ○ Sexual Health Services
- ○ Other (please specify)

Section 6: Referral and clinical management:

24. Can asylum seeker patients be referred to secondary care?
    - ○ Yes – All chronically infected patients
    - ○ Yes – A selection based on clinical indicators
    - ○ No
    - ○ Unsure
25. [If answered YES – A selection based on clinical indicators
    What are these clinical indicators?
    - ☐ Viral load
    - ☐ HBe antigen status
    - ☐ ALT
    - ☐ Unsure
    - ☐ Other (please specify)
26. Which services can refer asylum seeker patients to secondary care? Select all those that apply.
    - ☐ Health care service at receiving centre/national border control
    - ☐ Maternity units
    - ☐ General Practice
    - ☐ Public health services/health protection units
    - ☐ Hospitals/clinics
    - ☐ Sexual health services
    - ☐ Unsure
    - ☐ Other (please specify)

Section 7: Contact tracing and vaccination:

27. Is **hepatitis B screening/testing** offered to **contacts** (household and/or sexual contacts) of asylum seekers **hepatitis B** positive patients?
    - ○ No
    - ○ Yes – all contacts
    - ○ Yes – a selection of contacts (please specify)
    - ○ Unsure
28. [If hepatitis YES to HBV screening offered to contacts of asylum seeker hepatitis B positive patients]
    Are **hepatitis B negative contacts** (household and/or sexual contacts) of hepatitis B positive patients **vaccinated**?
    - ○ No
    - ○ Yes – all hepatitis B negative contacts
    - ○ Yes – a selection of hepatitis B negative contacts (please specify)
    - ○ Unsure

29. [If YES to vaccination of hepatitis B negative contacts of hepatitis B positive patients]
    Who has the main responsibility for the vaccination of contacts?
    - General Practitioners
    - Public health services/health protection units
    - Hospitals/clinics
    - Sexual Health Services
    - Hospital/clinics
    - Other (please specify)
    - Unsure
30. Is **hepatitis C screening/testing** offered to **contacts** (household and/or sexual contacts) of asylum seekers **HCV** positive patients?
    - No
    - Yes – all contacts
    - Yes –a selection of contacts (please specify)
    - Unsure
31. [If YES to hepatitis B or C screening/testing offered to contacts of asylum seeker hepatitis positive patients]

Who has the main responsibility for screening/testing of contacts for hepatitis B or hepatitis C positive patients for other hepatitis viruses?
- Health care service at receiving centres/national border control
- General Practicitioners
- Public health services/health protection units
- Sexual Health Services
- Hospitals/clinics
- Other (please specify)
- Unsure

Section 8: Professional practice and training:

32. How common is it for **asylum seeker medical care services to have the following available:**

	Very common	Variable or not routinely	Rarely or never	Unsure
Materials about viral hepatitis in the national language				
Materials about viral hepatitis in other languages				
Interpreter services via a telephone				
Face to face interpreter services				

33. Is training available for staff working with asylum seekers to improve their knowledge and skills in identifying and dealing with viral hepatitis?
    - Yes (please give details)
    - No
    - Unsure

    Make a comment on your choice here ______________________________

Section 8: Professional practice and training for Hepatitis B:

34. Are there any official national guidelines **about hepatitis B** screening and patient management in place in your country? If yes please give name and publisher.

Guidelines	
☐ General hepatitis B guidelines	

☐ Specific guidelines for refugees and asylum seekers	
☐ Other hepatitis B guideline	

35. [If there are official national guidelines about hepatitis B screening and patient management in place in your country]
    Does this specific hepatitis B guideline for refugee and asylum seekers include information about:
    - ☐Clinical indications and risk factors to prompt a test for hepatitis B
    - ☐Pre-test information and advice for patients
    - ☐Disease-related advice and guidance (counselling) for patients following positive diagnosis
    - ☐Onward referral of chronic hepatitis B patients to other services
    - ☐Recommendations to tailor services or information for people from a migrant or ethnic minority background
    - ☐Assessment of liver disease
    - ☐Treatment strategies

Section 8: Professional practice and training for Hepatitis C:

36. Are there any official national guidelines in place in your country **about hepatitis C** screening and patient management? If yes please give name and publisher.

Guidelines	
☐ General hepatitis C guidelines	
☐ Specific guidelines for refugees and asylum seekers	
☐ Other hepatitis C guideline	

37. [If YES to official national guidelines about hepatitis C screening and patient management in place in your country]
    Does this specific hepatitis C guideline for refugee and asylum seekers include information about:
    - ☐Clinical indications and risk factors to prompt a test for hepatitis C
    - ☐Pre-test information and advice for patients
    - ☐Disease-related advice and guidance (counselling) for patients following positive diagnosis
    - ☐Onward referral of chronic hepatitis B patients to other services
    - ☐Recommendations to tailor services or information for people from a migrant or ethnic minority background
    - ☐Assessment of liver disease
    - ☐Treatment strategies

Section 9: Barrier questions:

Uptake of screening among at risk groups

38. To what extent do you agree with the following statements as explanations of the current low uptake of hepatitis B and C screening among people from a migrant or ethnic minority background in your country.

**Statements**	**Strongly disagree**	**Disagree**	**Neither agree nor disagree**	**Agree**	**Strongly agree**

Limited awareness and knowledge about hepatitis B and C in general (including the ways of transmission) and their consequences (e.g. the link to liver cancer)					
Subjective feeling of being healthy and hence unlikely to be infected with hepatitis B/C					
First generation migrants from hepatitis B and C endemicity countries are not aware that they have a significantly higher risk of being infected with hepatitis B/C					
Limited awareness that screening and subsequent treatment can prevent future complications					
Fear of social stigma and discrimination if found to be hepatitis B/C positive (e.g. fear of losing job)					
Lack of information about where to go for a test					
Lack of access to free/affordable health care					
Language barriers when visiting health services (limited availability of translated materials or interpreter services)					

39. If you think there are other explanations, please give details in the box below.

Screening offered by primary health care provides

40. To what extent do you agree with the following statements as explanations of why migrants are not being screened/tested for hepatitis B/C at the point of first contact with primary health care services/GPs in your country.

**Statements**	**Strongly disagree**	**Disagree**	**Neither agree nor disagree**	**Agree**	**Strongly agree**
Health services are unable to identify patients with migration-related risk factors (such as country of birth or ethnic origin) as this data is not routinely collected					
Primary care providers/GPs are not aware that migrants from hepatitis B and C endemic countries have a significantly higher risk and should be offered screening					
Patients refuse testing despite primary care providers/GPs offering screening					
Limited awareness among primary health care providers/GPs about the scope of new, improved anti-viral treatments that can potentially cure the disease or significantly reduce disease progression					
Primary care providers/GPs rarely have translated materials about viral hepatitis or interpreter services available for patients					

Hepatitis screening of asymptomatic risk groups is generally not covered under the general health care service/insurance scheme in my country					
There is limited guidance available to primary health care professionals/GPs on screening for viral hepatitis among at risk groups					
Health care professionals/GPs do not have time to offer screening					

41. If you think there are other explanations, please give details in the box below.

Disease-related counselling, onward referral and clinical management of hepatitis B/C patients.

42. To what extent do you agree with the following statements as explanations of why hepatitis B/C cases do not reach specialized health care (e.g. hepatologists) for further investigation and treatment in your country.

**Statements**	**Strongly disagree**	**Disagree**	**Neither agree nor disagree**	**Agree**	**Strongly agree**
In antenatal screening programmes the focus is mainly on vaccination of the baby of hepatitis B positive mothers, not on hepatitis care for the women testing positive					
Time constraints affect health care professionals/GPs ability to provide patients with disease-related counselling and referral advice					
Some health care services are not reimbursed for providing disease-related counselling and referral advice to patients					
Newly diagnosed patients generally do not receive comprehensive counselling on the consequences of the disease, treatment options and referral, and hence do not seek specialist care					
There are too few specialists to whom the patients can be referred to for specialized care					
The antiviral treatment itself is generally not covered under the general health care service/insurance scheme in my country					
Patients are referred to the specialist but refuse further investigation or treatment					
There is limited guidance available to primary health care professionals about onward referral, counselling and patient management of hepatitis B/C patients					
Although training on viral hepatitis management is available for health care providers, uptake is generally low among professionals.					

Patients from a migrant or ethnic minority background face language barriers when visiting health services (limited availability of translated materials or interpreter services)					

43. If you think there are other explanations, please give details in the box below:

Comments

44. If you have any comments in general or regarding the screening, counseling, referral and/or treatment of Hepatitis B/C in your country, we would be grateful to learn more and benefit from your experience. Please write any remarks in the text box below.

Thank you for completing this survey.
