# Supplementary material for: Language support for linguistic minority chronic hepatitis B/C patients: an exploratory study of availability and clinicians’ perceptions of language barriers in six European countries
Source: BMC Health Serv Res. 2017 Feb 20;17:150. doi: 10.1186/s12913-017-2095-5 (PMC5319068; doi:10.1186/s12913-017-2095-5)
Supplement: Additional file 4: — Screening for viral hepatitis in Sexual Health Services/by Genit-Urinary Medicine specialist clinics survey. (PDF 344 kb) [file 12913_2017_2095_MOESM4_ESM.pdf]

Screening for viral hepatitis in Sexual Health Services / by Genito-Urinary Medicine (GUM) specialist clinics.

Welcome text

You have been approached to complete this survey as an expert in your professional field of sexual health care, and/or as part of your involvement in a national or regional level organisation that represents clinicians and/or public health professionals. It is from this representative position that we would like you to respond to the survey. We are interested in the general or routine viral hepatitis related screening and clinical management practices in your country.

This survey has been sent to experts in seven different countries in the EU. We recognise that practices will differ between the seven countries and have tried to reflect this diversity in the structure and answering options. Although there may be aspects of the survey that are not applicable to the situation in your country, the structure of the survey will enable you to reflect that in your answers.

The survey will take approximately 15-20 minutes. It is possible to stop and complete the survey at a later time.

Please be assured that your responses will be kept anonymous

Section 1: Respondent profile

These questions relate to your involvement in a national or regional level organisation that represents clinicians and/or public health professionals and/or patients.

1. What is the name of your organisation? ……………..

2. What type of organisation is it?
   - NGO
   - National Government
   - Regional Government
   - Clinical Association
   - Professional Organisation
   - University
   - Patient association
   - Other (please specify): ……….

3. What is your Job Role/Job Title: ………..

4. Do you also have clinical responsibilities and are directly involved in the care of patients?
   - Yes
   - No [skip to section 2]

5. [If YES to have clinical responsibilities and to be directly involved in the care of patients] What type of medical facility do you work in?
   - GP practice
   - Public health service/health protection unit
   - Clinic (outside a hospital)
   - General hospital

- University/Teaching hospital
- Health care service at receiving center/national border control

6. [If YES to have clinical responsibilities and to be directly involved in the care of patients]
   What is your medical specialism/clinical role?
   - General Practitioner
   - Infectious Disease specialist
   - Gastroenterologist/Hepatologist
   - Gynaecologist/Obstetrician
   - Other (please specify): …………

7. [If YES to have clinical responsibilities and to be directly involved in the care of patients]
   How often do you see patients with a chronic hepatitis B or hepatitis C infection?
   - Never
   - A few patients per year (1-10)
   - On a monthly basis
   - On a weekly basis

Section 2: Indications/risks factors for screening:

8. How common is it for professionals **in your speciality** to test for **hepatitis B** in the following circumstances?

	**Very common**	**Variable or not routinely**	**Rarely or never**	**Unsure**
A request from a patient concerned that they may have been exposed				
Migrants from hepatitis B endemic areas				
Injecting Drug Users (IDUs)				
Sex workers				
Homosexual men (MSM)				
HIV positive patients				
Hepatitis C positive patients				

9. [If 'VERY COMMON' or 'VARIABLE OR NOT ROUTINELY' to **screening** of any subgroups was selected]
   After screening, are **hepatitis B negative** individuals **vaccinated**?

	**Yes**	**Sometimes**	**No**	**Unsure**
A request from a patient concerned that they may have been exposed				
Migrants from hepatitis B endemic areas				
Injecting Drug Users (IDUs)				
Sex workers				
Homosexual men (MSM)				
HIV positive patients				
Hepatitis C positive patients				

10. [If YES or SOMETIMES to **vaccination** of hepatitis B negative individuals was selected]
    Is **individual co-payment/contribution** required for vaccination?

	Yes	No	Unsure
A request from a patient concerned that they may have been exposed			
Migrants from hepatitis B endemic areas			
Injecting Drug Users (IDUs)			
Sex workers			
Homosexual men (MSM)			
HIV positive patients			
Hepatitis C positive patients			

11. How common is it for professionals in your speciality to test for **hepatitis C** in the following circumstances?

	Very common	Variable or not routinely	Rarely or never	Unsure
A request from a patient concerned that they may have been exposed				
Migrants from hepatitis C endemic areas				
Injecting Drug Users (IDUs)				
Sex workers				
Homosexual men (MSM)				
HIV positive patients				
Hepatitis C positive patients				

12. [If ‘VERY COMMON’ or ‘VARIABLE OR NOT ROUTINELY’ to **screening** of any subgroups was selected] Is **individual co-payment/contribution** required for **hepatitis B or C screening** from migrants from hepatitis B or C endemic areas?
    - Yes – contribution required from all
    - No – free for all
    - Only free for some (please indicate for which subgroups co-payment is not required)
    - Unsure

Section 3: Pre-test information and advice (counselling) before testing:

Content of pre-test information

13. How common is it for **sexual health services/professionals in your speciality** to provide information and advice on the following topics **before testing** a patient for hepatitis B:

	Very common	Variable or not routinely	Rarely or never	Unsure
General information about the virus				
The test itself				
Implications of a positive test				
Future effect on relationships / social network				
Support available to patient				
Treatment options				
Modes of transmission				

Content of pre-test information

14. How common is it for **sexual health services/professionals in your speciality** to provide information and advice on the following topics **before testing** a patient for hepatitis C:

	**Very common**	**Variable or not routinely**	**Rarely or never**	**Unsure**
General information about the virus				
The test itself				
Implications of a positive test				
Future effect on relationships / social network				
Support available to patient				
Treatment options				
Modes of transmission				

Section 4: Part 1: Disease-related advice and guidance (counselling) following a positive result:

15. Who has the **main responsibility** for providing **disease-related counselling/advice and guidance** to patients following a positive diagnosis for viral hepatitis?
    - Sexual health services/GUM clinics
    - General practitioners
    - Public health services/health protection units
    - Infectious Disease specialists (not in public health services/heath protection units)
    - Specialists (e.g. Gastroenterologists/Hepatologists)
    - Other (please specify)
    - Unsure

16. Are sexual health services/GUM clinics involved in the care of hepatitis B and/or hepatitis C positive patients for any of the following:

	**Yes**	**No**	**Unsure**
Disease-related advice and guidance (counselling)			
Screening/testing for other hepatitis viruses			
Contact tracing			
Patient management			

Section 4: Part 2: Content of disease-related advice and guidance (counselling):

17. How common is it for **sexual health services** to include the following topics in disease-related advice and guidance (counselling):

	Very common	Variable or not routinely	Never	Unsure
General information about the disease				
Hygiene measures to prevent transmission to others				
Contact tracing				
Other tests required				
What to expect, onward referral				
Treatment options, benefits and side effects				
The importance of a healthy lifestyle, especially the damaging role of alcohol consumption				
Mental health promotion and staying positive				

Patient organisations / support groups				

Section 5: Referral and clinical management:

18. Which hepatitis B or hepatitis C positive patients are referred by sexual health services to specialist care for chronic viral hepatitis?
    - o All patients
    - o A selection based on clinical indicators
    - o None – referral to specialist care is via another service
19. [If answered a selection based on clinical indicators to question 19]
    What are these clinical indicators?
    - ☐ Viral load
    - ☐ HBe antigen status
    - ☐ ALT
    - ☐ Unsure
    - ☐ Other (please, specify)
20. Who can sexual health services refer chronic viral hepatitis cases to?
    - o Directly to specialist secondary care
    - o Referral to specialist secondary care via GP
    - o Referral to specialist secondary care via another service (please give details)
    - o Unsure
21. [If answered referral is via another service]
    If referral to specialist secondary care is via another service, please give details.

Section 6: Contact tracing and vaccination:

22. Is **hepatitis B screening/testing** offered to **contacts** (**household and/or sexual)** of hepatitis B positive patients?
    - o No
    - o Yes – all contacts
    - o Yes – a selection of contacts (please specify)
    - o Unsure
23. [If YES to vaccination offered to hepatitis B negative contacts of hepatitis B positive patients]
    Are **hepatitis B negative contacts** (household and/or sexual contacts) of **hepatitis B positive** patients vaccinated?
    - o No
    - o Yes – all hepatitis B negative contacts
    - o Yes – a selection of hepatitis B negative contacts (please specify)
    - o Unsure

24. [If YES to vaccination of hepatitis B negative contacts of hepatitis B positive patients]
    Who has the main responsibility for the vaccination of contacts?
    - o General Practitioners
    - o Public health services/health protection units
    - o Sexual Health Services

- Hospitals/clinics
- Other

25. Is **hepatitis C screening/testing** offered to **contacts** (household and/or sexual contacts) **of hepatitis C positive patients**?
    - No
    - Yes – all contacts
    - Yes – a selection of contacts (please specify)
    - Unsure
26. [If YES to hepatitis B screening offered to contacts of hepatitis C positive patients]
    Who has the main responsibility for the screening/testing of contacts for hepatitis B and C?
    - General Practitioners
    - Public health services/health protection units
    - Sexual Health Services
    - Hospitals/clinics
    - Other
    - Unsure

Section 7: Professional practice and training:

27. How common is it for sexual health services/GUM clinics to have the following available for patients

	Very common	Variable or not routinely	Rarely or never	Unsure
Materials about viral hepatitis in the national language				
Materials about viral hepatitis in other languages				
Interpreter services via a telephone				
Face to face interpreter services				

28. Is training available for sexual health service staff to improve their knowledge and skills in viral hepatitis?
    - Yes (please give details)
    - No
    - Unsure

Make a comment on your choice here:

Section 7: Professional practice and training for hepatitis B:

29. Are there any official national guidelines about **Hepatitis B** screening and patient management in place in your country? If yes, please give name and publisher:
    - ☐ General Hepatitis B guidelines: ………………
    - ☐ Specific guidelines for sexual health services/GUM clinics: ………………
    - ☐ Other hepatitis B guidelines: ………………

30. [If there are official national guidelines about **Hepatitis B** screening and patient management in place in your country]

Does this specific guideline for sexual health services/GUM clinics include information about:

- ☐ Clinical indications and risk factors to prompt a test for hepatitis B
- ☐ Pre-test information and advice for patients
- ☐ Disease-related advice and guidance (counselling) for patients following positive diagnosis
- ☐ Onward referral of chronic viral hepatitis patients to other services
- ☐ Recommendations to tailor services or information for people from a migrant or ethnic minority background
- ☐ Assessment of liver disease
- ☐ Treatment strategies

Section 7: Professional practice and training for Hepatitis C:

31. Are there any official national guidelines about **Hepatitis C** screening and patient management in place in your country? If yes, please give name and publisher.
    - ☐ General Hepatitis C guidelines: ………………
    - ☐ Specific guidelines for sexual health services/GUM clinics: ………………
    - ☐ Other hepatitis C guidelines: ………………

32. [If there are official national guidelines about **Hepatitis C** screening and patient management in place in your country]
    Does this specific guideline for sexual health services/GUM clinics include information about:

    - ☐ Clinical indications and risk factors to prompt a test for hepatitis C
    - ☐ Pre-test information and advice for patients
    - ☐ Disease-related advice and guidance (counselling) for patients following positive diagnosis
    - ☐ Onward referral of chronic viral hepatitis patients to other services
    - ☐ Recommendations to tailor services or information for people from a migrant or ethnic minority background
    - ☐ Assessment of liver disease
    - ☐ Treatment strategies

Section 8: Barrier questions:

Uptake of screening among at risk groups

33. To what extent do you agree with the following statements as explanations of the current low uptake of hepatitis B and C screening among people from a migrant or ethnic minority background in your country.

**Statements**	**Strongly disagree**	**Disagree**	**Neither agree nor disagree**	**Agree**	**Strongly agree**
Limited awareness and knowledge about hepatitis B and C in general (including the ways of transmission) and their consequences (e.g. the link to liver cancer)					
Subjective feeling of being healthy and hence unlikely to be infected with hepatitis B/C					
First generation migrants from hepatitis B and C endemic countries are not aware that they have a					

significantly higher risk of being infected with hepatitis B/C					
Limited awareness that screening and subsequent treatment can prevent future complications					
Fear of social stigma and discrimination if found to be hepatitis B/C positive (e.g. fear of losing job)					
Lack of information about where to go for a test					
Lack of access to free/affordable health care					
Language barriers when visiting health services (limited availability of translated materials or interpreter services)					

34. If you think there are other explanations, please give details in the box below.

Screening offered by primary health care provides

35. To what extent do you agree with the following statements as explanations of why migrants are not being screened/tested for hepatitis B/C at the point of first contact with primary health care services/GPs in your country.

**Statements**	**Strongly disagree**	**Disagree**	**Neither agree nor disagree**	**Agree**	**Strongly agree**
Health services are unable to identify patients with migration-related risk factors (such as country of birth or ethnic origin) as this data is not routinely collected					
Primary care providers/GPs are not aware that migrants from hepatitis B and C endemic countries have a significantly higher risk and should be offered screening					
Patients refuse testing despite primary care providers/GPs offering screening					
Limited awareness among primary health care providers/GPs about the scope of new, improved anti-viral treatments that can potentially cure the disease or significantly reduce disease progression					
Primary care providers/GPs rarely have translated materials about viral hepatitis or interpreter services available for patients					
Hepatitis screening of asymptomatic risk groups is generally not covered under the general health care service/insurance scheme in my country					
There is limited guidance available to primary health care professionals/GPs on screening for viral hepatitis among at risk groups					

Health care professionals/GPs do not have time to offer screening					

36. If you think there are other explanations, please give details in the box below.

Disease-related counselling, onward referral and clinical management of hepatitis B/C patients.

37. To what extent do you agree with the following statements as explanations of why hepatitis B/C cases do not reach specialized health care (e.g. hepatologists) for further investigation and treatment in your country.

Statements	Strongly disagree	Disagree	Neither agree nor disagree	Agree	Strongly agree
In antenatal screening programmes the focus is mainly on vaccination of the baby of hepatitis B positive mothers, not on hepatitis care for the women testing positive					
Time constraints affect health care professionals/GPs ability to provide patients with disease-related counselling and referral advice					
Some health care services are not reimbursed for providing disease-related counselling and referral advice to patients					
Newly diagnosed patients generally do not receive comprehensive counselling on the consequences of the disease, treatment options and referral, and hence do not seek specialist care					
There are too few specialists to whom the patients can be referred to for specialized care					
The antiviral treatment itself is generally not covered under the general health care service/insurance scheme in my country					
Patients are referred to the specialist but refuse further investigation or treatment					
There is limited guidance available to primary health care professionals about onward referral, counselling and patient management of hepatitis B/C patients					
Although training on viral hepatitis management is available for health care providers, uptake is generally low among professionals.					
Patients from a migrant or ethnic minority background face language barriers when visiting health services (limited availability of translated materials or interpreter services)					

38. If you think there are other explanations, please give details in the box below.

Comments

39. If you have any comments about the survey in general or regarding the screening, counselling, referral and/or treatment of Hepatitis B/C in your country, we would be grateful to learn more and benefit from your experience. Please write any comments in the text box below.

Thank you for completing this survey.
