# Supplementary material for: Language support for linguistic minority chronic hepatitis B/C patients: an exploratory study of availability and clinicians’ perceptions of language barriers in six European countries
Source: BMC Health Serv Res. 2017 Feb 20;17:150. doi: 10.1186/s12913-017-2095-5 (PMC5319068; doi:10.1186/s12913-017-2095-5)
Supplement: Additional file 5: — Specialist secondary care survey. (PDF 359 kb) [file 12913_2017_2095_MOESM5_ESM.pdf]

Patient Management and Treatment in Secondary Care

Welcome text
You have been approached to complete this survey as an expert in your professional field of viral hepatitis care, and/or as part of your involvement in a national or regional level organisation that represents clinicians and/or public health professionals. It is from this representative position that we would like you to respond to the survey. We are interested in the general or routine viral hepatitis related screening and clinical management practices in your country.

This survey has been sent to experts in seven different countries in the EU. We recognise that practices will differ between the seven countries and have tried to reflect this diversity in the structure and answering options. Although there may be aspects of the survey that are not applicable to the situation in your country, the structure of the survey will enable you to reflect that in your answers.

The survey will take approximately 15 minutes. It is possible to stop and complete the survey at a later time.

Please be assured that your responses will be kept anonymous.

Section 1: Respondent profile

These questions relate to your involvement in a national or regional level organisation that represents clinicians and/or public health professionals and/or patients.

1. What is the name of your organisation? …………….

2. What type of organisation is it?
   - o NGO
   - o National Government
   - o Regional Government
   - o Clinical Association
   - o Professional Organisation
   - o University
   - o Patient association
   - o Other (please specify): ……….

3. What is your Job Role/Job Title: ………..

4. Do you also have clinical responsibilities and are directly involved in the care of patients?
   - o Yes
   - o No [skip to section 2]

5. [If YES to have clinical responsibilities and to be directly involved in the care of patients]
   What type of medical facility do you work in?
   - o GP practice
   - o Public health service/health protection unit
   - o Clinic (outside a hospital)
   - o General hospital
   - o University/Teaching hospital

- Health care service at receiving center/national border control

6. [If YES to have clinical responsibilities and to be directly involved in the care of patients]
   What is your medical specialism/clinical role?
   - General Practitioner
   - Infectious Disease specialist
   - Gastroenterologist/Hepatologist
   - Gynaecologist/Obstetrician
   - Other (please specify): …………

7. [If YES to have clinical responsibilities and to be directly involved in the care of patients]
   How often do you see patients with a chronic hepatitis B or hepatitis C infection?
   - Never
   - A few patients per year (1-10)
   - On a monthly basis
   - On a weekly basis

Section 2: Referral to secondary care and disease-related advice and guidance (counselling):

8. In your experience, how common is it for professionals in your speciality to receive their hepatitis B/C patients from the following settings/specialities. If there are other settings/specialities, please give details.

	Very common	Variable or not routinely	Rarely or never	Unsure
General Practitioner				
Public health services/health protection units				
Sexual Health Services/Genito-urinary medicine clinics				
Midwives/Obstetrician/Gynaecologist				
Asylum seeker service				
Infectious Disease specialists				
Referral from an outreach screening programme				
Referral from centres testing injecting drug users				

9. If there are other settings/specialities, please give details.

10. Who has the **main responsibility** for providing disease-related **advice and guidance (counselling)** to patients following a positive diagnosis for viral hepatitis in your country?
    - General Practitioners
    - Public health services/health protection units
    - Infectious disease specialists (not in public health services/health protection units)
    - Specialists (e.g. Gastroenterologists/Hepatologists)
    - Other (please specify)

Section 3: Content of disease-related advice and guidance (counselling):

11. How common is it for **professionals in your speciality** to include the following topics in the consultation with a hepatitis B or C patient:

Consultation topics	Very common	Variable or not routinely	Rarely or never	Unsure
General information about the disease				
Hygiene measures to prevent transmission to others				
Contact tracing				
Other tests required				
Treatment options, benefits and side effects				
The importance of a healthy lifestyle, especially the damaging role of alcohol consumption				
Mental health promotion and staying positive				
Patient organisations / support groups				

Section 4: Screening/testing positive patients for other hepatitis viruses:

12. Are **hepatitis B positive patients** screened/tested for hepatitis C?
    - No
    - Yes – all patients
    - Yes – a selection of patients (please specify which)
    - Unsure
13. Are **hepatitis C positive patients** screened/tested for hepatitis B?
    - No
    - Yes – all patients
    - Yes – a selection of patients (please specify which)
    - Unsure
14. [If YES to screening/testing of hepatitis B/C positive patients for other viruses]
    Who has the **main responsibility** for screening/testing of **hepatitis B or hepatitis C positive patients** for other hepatitis viruses?
    - General Practitioners
    - Public health services/health protection units
    - Hospitals/clinics
    - Sexual Health Services
    - Other (please specify)
15. Are **hepatitis C positive patients** who are found to be hepatitis B negative **vaccinated** against hepatitis B?
    - No
    - Yes – all patients
    - Yes – a selection of patients (please specify)
    - Unsure
16. [If YES to hepatitis B vaccination of hepatitis C positive patients found to be hepatitis B negative]
    Who has the **main responsibility** for vaccination of **hepatitis C positive patients** found hepatitis B negative??
    - General Practitioners
    - Public health services/health protection units
    - Hospitals/clinics

- Sexual Health Services
- Other (please specify)

Section 5: Contact tracing and vaccination:

17. Is **hepatitis B screening/testing** offered to **contacts** (household and/or sexual contacts) of hepatitis B positive patients?
    - No
    - Yes –all contacts
    - Yes –a selection of contacts (please specify)
    - Unsure
18. Are **hepatitis B negative contacts** (household and/or sexual contacts) of hepatitis B positive patients vaccinated?
    - No
    - Yes –all hepatitis B negative contacts
    - Yes –a selection of hepatitis B negative contacts (please specify)
    - Unsure
19. [If YES to vaccination offered to hepatitis B negative contacts of hepatitis B positive patients] Who has responsibility for vaccination of **contacts**?
    - General Practitioners
    - Public health services/health protection units
    - Sexual Health Services
    - Hospitals/clinics
    - Other (please specify)
20. Is **hepatitis C screening/testing** offered to **contacts (household and/or sexual contacts) of hepatitis C positive patients**?
    - No
    - Yes –all contacts
    - Yes –a selection contacts (please specify)
    - Unsure
21. [If YES to screening/testing offered to contacts of hepatitis B/C positive patients] Who has the **main responsibility** for the screening/testing of contacts for hepatitis B and C?
    - General Practitioners
    - Public health services/health protection units
    - Sexual Health Services
    - Hospitals/clinics
    - Other (please specify)

Section 6: The role of the GP:

22. How common is it that the following patients would be **referred back to their GP** from specialist care?

Patient groups	Very common	Variable or not routinely	Rarely or never	Unsure
Those who do not qualify for treatment after the initial evaluation				
Those undergoing antiviral treatment				
Those who have sustained virological response				

due to treatment				
Those who are non responders to treatment				

23. How common is it that a **GP** would be involved in monitoring the following indicators in a patient **undergoing antiviral treatment**?

	Very common	Variable or not routinely	Rarely or never	Unsure
ALT levels				
Viral load				
Side effects				

Section 7: Diagnostics

24. How common is it for the following diagnostics to be used in the initial evaluation of a hepatitis B or C positive patient?

	Very common	Variable or not routinely	Rarely or never	Unsure
HBeAg and/or anti-HBe (for hepatitis B only)				
ALT				
Other biochemical markers (AST, GGT, serum albumin, etc)				
Quantitative viral load (hepatitis B DNA or hepatitis C RNA)				
Genotype				
Ultrasound				
Liver biopsy				
Transient elastography (e.g. fibroscan)				

Section 8: Treatment options:

25. Are there **limitations** on the use of the following antiviral drugs for **hepatitis B**?

HBV drugs	No limitations	Yes – some restrictions	Yes – totally limited / cannot be prescribed	Unsure
(Pegylated) Interferon alpha				
Lamivudine				
Telbivudine				
Adefovir				
Entecavir				
Tenofovir				

26. [If YES to some restriction on the use of Interferon alpha]
What are the **restrictions** for **Interferon alpha**?

- ☐ Can only be prescribed if resistance to another drug has developed
- ☐ Can only be prescribed for a limited duration

☐ Can only be prescribed by selected hospitals (e.g. tertiary centers)
☐ Can only be prescribed in selected geographic areas
☐ Other restrictions, please give details:

27. [If YES to some restriction on the use of Lamivudine]
What are the **restrictions** for **Lamivudine**?
☐ Can only be prescribed if resistance to another drug has developed
☐ Can only be prescribed for a limited duration
☐ Can only be prescribed by selected hospitals (e.g. tertiary centers)
☐ Can only be prescribed in selected geographic areas
☐ Other restrictions, please give details:

28. [If YES to some restriction on the use of Telbivudine]
What are the **restrictions** for **Telbivudine**?
☐ Can only be prescribed if resistance to another drug has developed
☐ Can only be prescribed for a limited duration
☐ Can only be prescribed by selected hospitals (e.g. tertiary centers)
☐ Can only be prescribed in selected geographic areas
☐ Other restrictions, please give details:

29. [If YES to some restriction on the use of Adefovir]
What are the **restrictions** for **Adefovir**?
☐ Can only be prescribed if resistance to another drug has developed
☐ Can only be prescribed for a limited duration
☐ Can only be prescribed by selected hospitals (e.g. tertiary centers)
☐ Can only be prescribed in selected geographic areas
☐ Other restrictions, please give details:

30. [If YES to some restriction on the use of Entecavir]
What are the **restrictions** for **Entecavir**?
☐ Can only be prescribed if resistance to another drug has developed
☐ Can only be prescribed for a limited duration
☐ Can only be prescribed by selected hospitals (e.g. tertiary centers)
☐ Can only be prescribed in selected geographic areas
☐ Other restrictions, please give details:

31. [If YES to some restriction on the use of Tenofovir]
What are the **restrictions** for **Tenofovir**?
☐ Can only be prescribed if resistance to another drug has developed
☐ Can only be prescribed for a limited duration
☐ Can only be prescribed by selected hospitals (e.g. tertiary centers)
☐ Can only be prescribed in selected geographic areas
☐ Other restrictions, please give details:

32. Are there **limitations** on the use of the following antiviral drugs for **hepatitis C**?

HCV drugs	No limitations	Yes – some restrictions	Yes – totally limited / cannot be prescribed	Unsure
(Pegylated) Interferon alpha				
Ribavirin				
Telaprevir				
Boceprevir				

33. [If YES to some restriction on the use of Interferon alpha]
What are the **restrictions** for **Interferon alpha**?
- ☐ Can only be prescribed if resistance to another drug has developed
- ☐ Can only be prescribed for a limited duration
- ☐ Can only be prescribed by selected hospitals (e.g. tertiary centers)
- ☐ Can only be prescribed in selected geographic areas
- ☐ Other restrictions, please give details:

34. [If YES to some restriction on the use of Ribavirin]
What are the **restrictions** for **Ribavirin**?
- ☐ Can only be prescribed if resistance to another drug has developed
- ☐ Can only be prescribed for a limited duration
- ☐ Can only be prescribed by selected hospitals (e.g. tertiary centers)
- ☐ Can only be prescribed in selected geographic areas
- ☐ Other restrictions, please give details:

35. [If YES to some restriction on the use of Telaprevir]
What are the **restrictions** for **Telaprevir**?
- ☐ Can only be prescribed if resistance to another drug has developed
- ☐ Can only be prescribed for a limited duration
- ☐ Can only be prescribed by selected hospitals (e.g. tertiary centers)
- ☐ Can only be prescribed in selected geographic areas
- ☐ Other restrictions, please give details:

36. [If YES to some restriction on the use of Boceprevir]
What are the **restrictions** for **Boceprevir**?
- ☐ Can only be prescribed if resistance to another drug has developed
- ☐ Can only be prescribed for a limited duration
- ☐ Can only be prescribed by selected hospitals (e.g. tertiary centers)
- ☐ Can only be prescribed in selected geographic areas
- ☐ Other restrictions, please give details:

37. Is **treatment** restricted for any of the following patient groups?

Patient groups	No restrictions	Some restrictions	Significant restrictions	Yes completely restricted	Unsure
Undocumented migrants without health care coverage or health insurance					
Patients without health care coverage or health insurance (not i)					
Asylum seekers (still in application procedure)					
Injecting drug users (current users)					
Patients abusing alcohol (current abusers)					

<u>Section 9: Professional practice and training:</u>

38. How common is it for professionals in your speciality to have the following available for patients:

	Very common	Variable or not routinely	Rarely or never	Unsure
Materials about viral hepatitis in the national language				
Materials about viral hepatitis in other languages				
Interpreter services via a telephone				
Face to face interpreter services				

39. Is training available for professionals in your speciality to improve their knowledge and skills in the clinical management of viral hepatitis?
   - ○ Yes (please give details)
   - ○ No
   - ○ Unsure

Make a comment on your choice here:

40. Is there a list of nationally certified centres/centres of excellence for the management and treatment of chronic hepatitis B and C?
   - ○ Yes (please give details)
   - ○ No
   - ○ Unsure

Make a comment on your choice here:

<u>Section 9: Professional practice and training for hepatitis B:</u>

41. Are there any official national guidelines about **Hepatitis B** screening and patient management in place in your country? If yes, please give name and publisher:
    - ☐ General Hepatitis B guidelines: ………………
    - ☐ Specific guidelines for specialists: ………………
    - ☐ Other hepatitis B guidelines: ………………

42. [If there are official national guidelines about Hepatitis B screening and patient management in your country]
    Does this specific guideline for specialists include information about:
    - ☐ Clinical indications and risk factors to prompt a test for hepatitis B
    - ☐ Pre-test information and advice for patients
    - ☐ Disease-related advice and guidance (counselling) for patients following positive diagnosis
    - ☐ Onward referral of chronic viral hepatitis patients to other services
    - ☐ Recommendations to tailor services or information for people from a migrant or ethnic minority background
    - ☐ Assessment of liver disease
    - ☐ Treatment strategies

<u>Section 9: Professional practice and training for hepatitis B:</u>

43. Are there any official national guidelines about **Hepatitis C** screening and patient management in place in your country? If yes, please give name and publisher:
    - ☐ General Hepatitis C guidelines: ………………
    - ☐ Specific guidelines for specialists: ………………
    - ☐ Other hepatitis C guidelines: ………………

44. [If there are official national guidelines about Hepatitis C screening and patient management in your country]
    Does this specific guideline for specialists include information about:
    - ☐ Clinical indications and risk factors to prompt a test for hepatitis C
    - ☐ Pre-test information and advice for patients
    - ☐ Disease-related advice and guidance (counselling) for patients following positive diagnosis
    - ☐ Onward referral of chronic viral hepatitis patients to other services
    - ☐ Recommendations to tailor services or information for people from a migrant or ethnic minority background
    - ☐ Assessment of liver disease
    - ☐ Treatment strategies

<u>Section 10: Barrier questions:</u>

Uptake of screening among at risk groups

45. To what extent do you agree with the following statements as explanations of the current low uptake of hepatitis B and C screening among people from a migrant or ethnic minority background in your country.

Statements	Strongly disagree	Disagree	Neither agree nor disagree	Agree	Strongly agree
Limited awareness and knowledge about hepatitis B and C in general (including the ways of transmission) and their consequences (e.g. the link to liver cancer)					
Subjective feeling of being healthy and hence unlikely to be infected with hepatitis B/C					
First generation migrants from hepatitis B and C endemicy countries are not aware that they have a significantly higher risk of being infected with hepatitis B/C					
Limited awareness that screening and subsequent treatment can prevent future complications					
Fear of social stigma and discrimination if found to be hepatitis B/C positive (e.g. fear of losing job)					
Lack of information about where to go for a test					
Lack of access to free/affordable health care					
Language barriers when visiting health services (limited availability of translated materials or interpreter services)					

46. If you think there are other explanations, please give details in the box below.

Screening offered by primary health care provides

47. To what extent do you agree with the following statements as explanations of why migrants are not being screened/tested for hepatitis B/C at the point of first contact with primary health care services/GPs in your country.

Statements	Strongly disagree	Disagree	Neither agree nor disagree	Agree	Strongly agree
Health services are unable to identify patients with migration-related risk factors (such as country of birth or ethnic origin) as this data is not routinely collected					
Primary care providers/GPs are not aware that migrants from hepatitis B and C endemic countries have a significantly higher risk and should be offered screening					
Patients refuse testing despite primary care providers/GPs offering screening					

Limited awareness among primary health care providers/GPs about the scope of new, improved anti-viral treatments that can potentially cure the disease or significantly reduce disease progression					
Primary care providers/GPs rarely have translated materials about viral hepatitis or interpreter services available for patients					
Hepatitis screening of asymptomatic risk groups is generally not covered under the general health care service/insurance scheme in my country					
There is limited guidance available to primary health care professionals/GPs on screening for viral hepatitis among at risk groups					
Health care professionals/GPs do not have time to offer screening					

48. If you think there are other explanations, please give details in the box below.

Disease-related counselling, onward referral and clinical management of hepatitis B/C patients.

49. To what extent do you agree with the following statements as explanations of why hepatitis B/C cases do not reach specialized health care (e.g. hepatologists) for further investigation and treatment in your country.

**Statements**	**Strongly disagree**	**Disagree**	**Neither agree nor disagree**	**Agree**	**Strongly agree**
In antenatal screening programmes the focus is mainly on vaccination of the baby of hepatitis B positive mothers, not on hepatitis care for the women testing positive					
Time constraints affect health care professionals/GPs ability to provide patients with disease-related counselling and referral advice					
Some health care services are not reimbursed for providing disease-related counselling and referral advice to patients					
Newly diagnosed patients generally do not receive comprehensive counselling on the consequences of the disease, treatment options and referral, and hence do not seek specialist care					
There are too few specialists to whom the patients can be referred to for specialized care					

The antiviral treatment itself is generally not covered under the general health care service/insurance scheme in my country					
Patients are referred to the specialist but refuse further investigation or treatment					
There is limited guidance available to primary health care professionals about onward referral, counselling and patient management of hepatitis B/C patients					
Although training on viral hepatitis management is available for health care providers, uptake is generally low among professionals.					
Patients from a migrant or ethnic minority background face language barriers when visiting health services (limited availability of translated materials or interpreter services)					

50. If you think there are other explanations, please give details in the box below.

<u>Section 11: Awareness raising/outreach campaigns:</u>

51. Are you aware of any campaigns or outreach programmes that aim to increase awareness of and promote screening for hepatitis B and/or C among migrant or ethnic minority groups in your country?

- o Yes (please give details below)
- o Not in detail/not personally but I know people who are aware of migrant-specific hepatitis screening programmes (please give details below)
- o No – I'm not aware of any
- o No – I know there are none

52. [if <u>Yes</u> or <u>Not in detail</u>: Option to give details for up to 5 campaigns]

Name and Job Role of Coordinator	
Organisation	
Contact details	
Name of the campaign:	
Nationality/ethnicity of target population(s)	
Setting(s) where target population are contacted (e.g. mosque, church, community centre, shops, online, home etc.)	
Year and duration (e.g. weeks/months/years/ongoing)	
Website	

Comments

53. If you have any comments about the survey in general or regarding the screening, counselling, referral and/or treatment of Hepatitis B/C in your country, we would be grateful to learn more and benefit from your experience. Please write any comments in the text box below.

Thank you for completing this survey.
